# Extensive Cutaneous Larva Migrans with Eczematous Reaction on Atypical Localization

**DOI:** 10.4269/ajtmh.15-0581

**Published:** 2016-06-01

**Authors:** Cristiane Comparin, Milena Marchini Rodrigues, Bruna Costa Santos

**Affiliations:** University of Mato Grosso do Sul, Mato Grosso do Sul, Brazil

**Figure 1.**
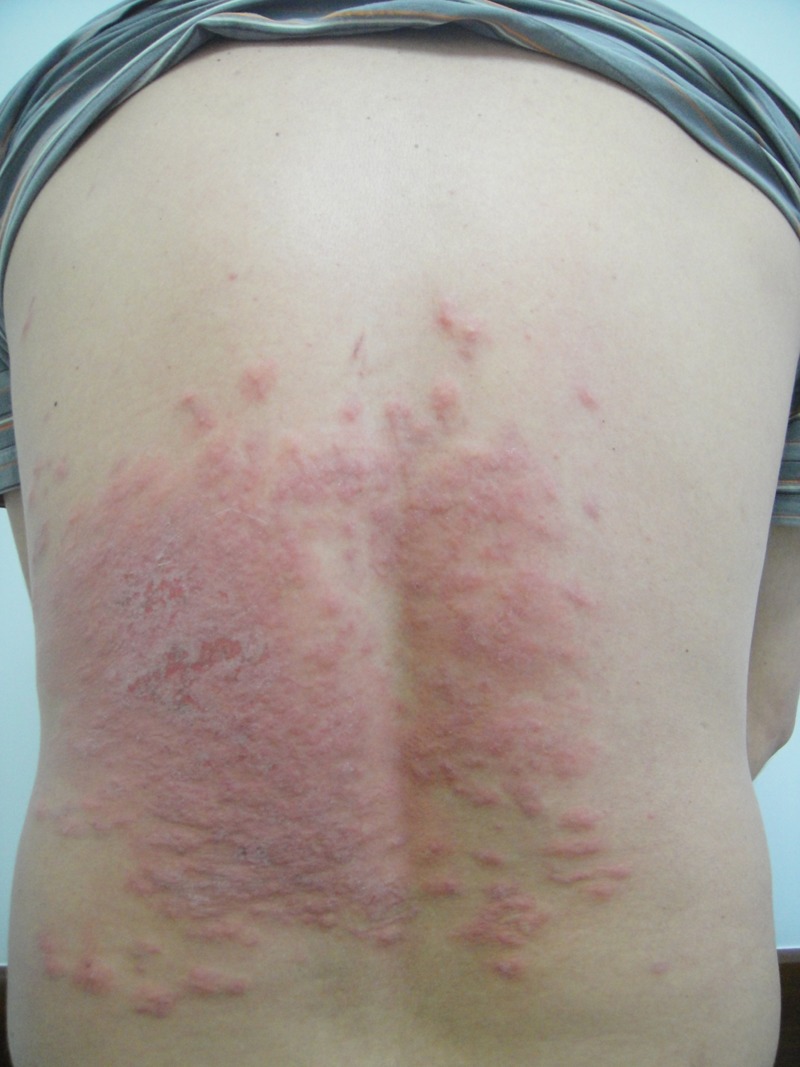
Large erythematous eczematous plaque on the lower half of patient's back (20 × 25 cm).

A 32-year-old man living in midwest Brazil presented with a 15-day history of skin eruption with severe itching after lying on a lawn in an amusement park for a few hours. The patient had no previous history of illness or use of medication. Clinical examination revealed a large (20 × 25 cm) erythematous eczematous plaque on the lower half of his back, with serpiginous tracks in some areas (Figures 1

and 2

**Figure 2.**
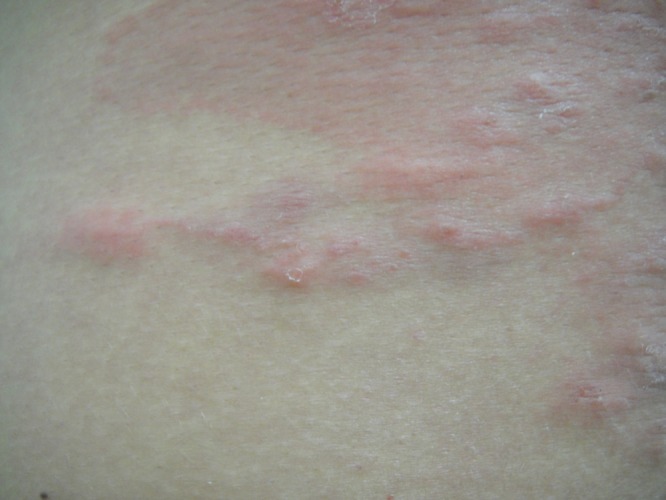
Serpiginous tracks in some areas of the erythematous eczematous plaque on the lower half of patient's back.

). The clinical characteristics of the lesions and epidemiological history are very suggestive for the diagnosis of cutaneous larva migrans (CLMs) with severe secondary eczematous reaction, on an atypical location. Therefore, considering the diagnosis, a treatment was prescribed with 5% thiabendazole, three times daily for 15 days, and 400 mg of oral albendazole daily for three consecutive days. The response to an antihelminthic drug and rapid resolution of the lesion supported the initial diagnosis of CLM.

CLM is caused by the accidental larval migration of nematodes (most commonly *Ancylostoma braziliense*) into the epidermis.^1^–^4^ This skin infestation, typical of subtropical and tropical regions, is endemic to the Caribbean, Central and South America, and Africa.^1^–^3^ With the growing frequency of travel to these areas, the incidence of the disease has also increased elsewhere. Walking barefoot on beach sand is a common feature.^2^

The larvae penetrate the corneal layer of human epidermis upon contact with soil contaminated with dog or cat feces containing excreted eggs, which in hot, humid environments become filariform larvae.^1^,^4^ These larvae migrate within the epidermis, causing an inflammatory reaction with intense itching, eruptions, and a serpiginous aspect that spreads a few centimeters a day.^1^–^4^ Lesions are usually few in number, and commonly affected sites include feet, thighs, and buttocks in adults.^1^,^5^ Only 7% of lesions occur on the trunk.^5^ The presence of multiple larvae may produce bizarre patterns.^5^ Diagnosis is clinical, based on the appearance of the lesions, with an emphasis on patient epidemiology.^1^,^2^ Biopsy is not useful.^1^–^3^ Differential diagnosis includes scabies, myiasis, tinea corporis, and contact dermatitis.^1^ Despite the self-limiting nature of the disease, severe itching, risk of secondary infection, and (in some reported cases) progression to Loeffler's syndrome warrant treatment with antihistamines and anthelminthics. First-line treatments are ivermectin and albendazole.^1^–^3^ In cases when oral drugs are contraindicated or not sufficient, topical application of 15% thiabendazole solution or ointments should be considered (not available in the United States or Canada).^1^–^3^ Treatment is simple, well tolerated, and effective.^1^–^3^
